# Synchronization of complex spatio-temporal dynamics with lasers

**DOI:** 10.1038/s41377-026-02198-5

**Published:** 2026-02-28

**Authors:** Jules Mercadier, Stefan Bittner, Marc Sciamanna

**Affiliations:** 1 Université de Lorraine, CentraleSupélec, LMOPS, Metz, France; 2 Chaire Photonique, LMOPS, Centralesupélec, Metz, France

**Keywords:** Nonlinear optics, Nonlinear optics

## Abstract

Synchronization is the spontaneous alignment of the dynamics of weakly-coupled oscillators. In addition to temporal dynamics like periodic and chaotic oscillations, also the spatio-temporal dynamics of spatially-extended systems like wildlife populations can synchronize. We exploit here the intrinsic spatio-temporal complex dynamics of broad area lasers to demonstrate such synchronization at lab-scale. Broad-area vertical-cavity surface-emitting lasers (BA-VCSELs) exhibit chaos from the nonlinear coupling between laser modes with different spatial profiles and polarization. When coupling two BA-VCSELs, several synchronization and anti-synchronization regimes are observed, highlighting the complex interplay between oscillating modes with different frequencies and spatial patterns. The correlation coefficient varies between 0.2 and 0.9 depending on the dynamics and on the time scale under analysis. Besides its fundamental interest, our experiment with commercial devices marks the first step towards real-world spatial multiplexing in multiple user physical-layer secure communication based on chaos synchronization.

## Introduction

Synchronization between coupled oscillators is a phenomenon encountered in a variety of fields including electronics^1,2^, chemical reactions^3^, biological networks^4^, and lasers^5–7^. In the simplest case of two coupled harmonic oscillators, synchronization means the two oscillators are frequency- and phase-locked, which was first observed for two coupled pendula^8^, and is usually described by the Adler equation^9^. Synchronization of clocks is of immense practical importance for time and frequency metrology.

More generally, synchronization means the ability of weakly coupled systems to develop correlated temporal dynamics. Synchronization can also occur between coupled chaotic systems, in spite of their inherent sensitivity to initial conditions^10–13^. Chaos synchronization is typically achieved with the master-slave unidirectional coupling configuration^14,15^, but also common-signal induced synchronization has been reported^16,17^. While temporal synchronization has been thoroughly explored, the extension to spatio-temporal systems remains largely unexplored. Systems with significant transverse extent can exhibit complex space- and time-dependent dynamics, but the spatial dimension introduces new challenges for achieving and understanding synchronization.

Synchronization of complex spatio-temporal dynamics occurs naturally for example in ecological systems^18,19^, the human brain^20^ and weather patterns^21^. Understanding and replicating these dynamics in artificial systems and at lab scale could revolutionize neuroscience, machine learning, biology and our understanding of complex systems in general.

Optical systems are an ideal testbed for studying nonlinear dynamics thanks to their compact size and fast time scales^11^. Complex spatio-temporal dynamics like pattern formation, filamentation^22,23^, and modal competition^24^ can be found for example in broad-area semiconductor lasers. In addition, theoretical studies have predicted the possibility to synchronize multimode and distributed optical systems^25,26^ and shown their potential for parallel chaotic information processing and multiple user encryption schemes^27,28^. Nonetheless, synchronization of complex spatio-temporal dynamics has been experimentally demonstrated so far only using a liquid crystal light valve with feedback^29^, and its implementation therefore remains far from practical physical-layer communication architectures.

We investigate the possibility to synchronize spatio-temporal chaotic systems using commercially available lasers in a table-top experiment. We take advantage of our recent discovery^30,31^ that broad area vertical cavity surface-emitting lasers (BA-VCSELs) naturally exhibit chaos involving nonlinear interactions between a large number of spatial modes with different polarization states^32–36^. By coupling two such lasers, we demonstrate synchronization in a controllable and reproducible way and analyze how synchronization relates to the spatial lasing modes. Besides its fundamental interest for the study of complex systems, our work marks the first step to a realistic implementation of spatial multiplexing for physical-layer secure communications at high bit rates.

**Fig. 1:**
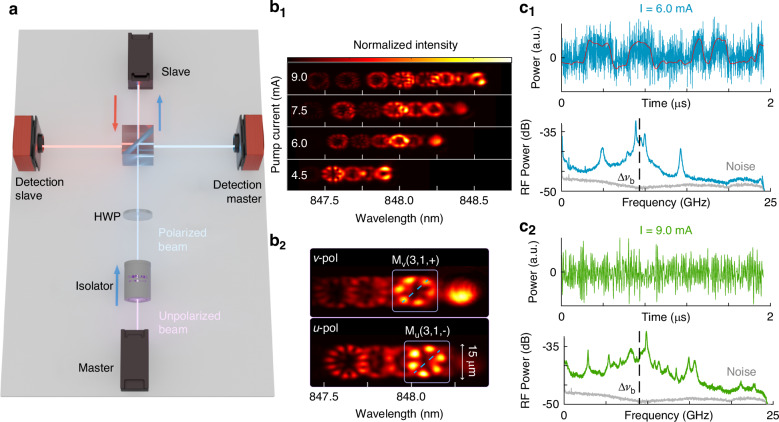
Setup and free-running master laser. **a** Schematic representation of the synchronization experiment setup. This configuration allows both the analysis of the dynamics of the master laser and the response of the slave laser under injection. It is possible to study both of the lasers in the spatio-spectral domain, using an imaging spectrometer (with a half-wave plate (HWP) to control the polarization orientation at the spectrometer input), or in the temporal domain, using two high-bandwidth photodetectors connected to a high-bandwidth real-time oscilloscope. **b**_1_ Spatio-spectral images of the free-running master laser for different pump currents in *u*-polarization, which is selected for injection; **b**_2_ Spatio-spectral image in both *u*- and *v*-polarizations for *I* = 6.5 mA, with the strong modes *M*_*u*,*v*_(3, 1) highlighted. The symmetry axis of the transverse mode is given by the blue dashed line to indicate the + (-) orientation of the modes. Master laser dynamics for *I*_*M*_= 6 mA (**c**_1_) and *I*_*M*_= 9 mA (**c**_2_) in the time and frequency domains. The vertical dashed lines in the RF-spectra indicate the birefringence Δ*ν*_*b*_. The red time trace in (**c**_1_) is low-pass filtered at 0.1 GHz

Specifically, we investigate two coupled BA-VCSELs which exhibit chaotic dynamics in free-running operation (see Fig. 1). Via the pump current, we can change the number of transverse lasing modes and the dynamical regimes^30,31^. Synchronization of chaotic dynamics is demonstrated for weak coupling via unidirectional optical injection. Remarkably, synchronization emerges despite significant differences in the spatial mode structures. It is mediated by the spectral alignment of the strong transverse modes of the master laser, defined here as those containing a significant portion of its total power. This reveals a mechanism in which frequency alignment prevails over spatial matching in enabling chaos synchronization in multimode systems. Spatial complexity, instead of preventing synchronization, therefore coexists with robust collective dynamics under appropriate coupling conditions.

We propose to investigate the effect of unidirectional optical injection between two BA-VCSELs in two distinct dynamical regimes: a first regime involving slow polarization-hopping dynamics, and a second regime featuring broadband chaotic dynamics.

## Results

We experimentally study unidirectional optical injection of one BA-VCSEL into another BA-VCSEL of the same model and analyze the dynamical response of the slave laser to the signal of the master laser. A simplified schematic of the experimental setup is shown in Fig. 1a, and a detailed description is provided in Supplementary Section 1. We begin with a summary of the properties of the master laser (see also refs. ^30,31^), and a comparison to the slave laser is presented in Supplementary Section 2.

The free-running BA-VCSELs lase in several transverse modes and two linear orthogonal polarization states, denoted by *u* and *v*. The dominant polarization depends on the pump current and changes at several polarization-switching points (PSPs, see Supplementary Fig. S2)^30^. The birefringence splitting between *u*- and *v*- polarization modes is around Δ*ν*_*b*_ ≈ 9 GHz (see Supplementary Section 2), and we refer to “red" (*u*) and “blue" (*v*) polarization according to their relative position in the optical spectrum.

Figure 1b shows spatio-spectral images measured with an imaging spectrometer (see Supplementary Section 1) for different pump currents. With increasing pump current, more transverse modes are progressively excited. A transverse mode with mode indices (*m*, *n*) has 2*m* intensity maxima in azimuthal direction and *n* maxima in radial direction. Furthermore, for *m* > 0, two distinct spatial orientations are possible, which we denote with + ( − ) when they are (anti-) symmetric with respect to a symmetry axis as shown in Fig. 1b_2_. In the following we denote the modes with *X*(*m*, *n*, ± ) where *X* ∈ {*M*, *S*} refers to the master and slave laser, respectively. It should be noted that modes (*m*, *n*, + ) and (*m*, *n*, − ) are not necessarily degenerate^37^, but can exhibit a splitting Δ*ν*_*O*_ of the order of several GHz (see Supplementary Section 2 A d). The actual splitting seems to depend on the transverse mode. Typically, only one of the orientations is excited for a given polarization, and the corresponding mode in the other polarization has the opposite orientation due to gain competition^30,38–40^. This is exemplified in Fig. 1b_2_ with the modes *M*_*v*_(3, 1, + ) and *M*_*u*_(3, 1, − ). However, in some cases both orientations of a transverse mode (*m*, *n*) lase in the same polarizations as shown in Supplementary Fig. S5.

The competition of lasing modes with different spatial profiles and polarizations results in rich nonlinear dynamics, as shown in Fig. 1c. As the pump current increases, the system undergoes a sequence of bifurcations that progressively enrich the temporal behavior^30,31^: initially periodic oscillations transition to quasi-periodic regimes, and eventually to chaotic dynamics (see Supplementary Fig. S6). These bifurcations are usually accompanied by a redistribution of power between different transverse modes and in some cases polarization switching points^30^. We restrict our analysis to parameter regions in which the master laser features chaotic dynamics for a focused investigation of synchronization in the presence of intrinsic multimode chaos.

The lasing dynamics features different time scales: the dominant frequencies are around the birefringence Δ*ν*_*b*_ [see Fig. 1c]. However, in some current regimes like around 6 mA, this fast dynamics coexists with a slower polarization-hopping dynamics with frequencies of the order of 100 MHz [see Fig. 1c_1_ and Supplementary Fig. S4], similar to observations for single-mode VCSELs^41–44^.

Next, we implement optical injection between two BA-VCSELs to investigate synchronization between them. Although the two lasers are of the same model, some differences in their spectral and dynamical properties inevitably remain. The comparison of their optical spectra and dynamics in Supplementary Section 2 B shows that spectral alignment can be achieved by adjusting the detuning via the temperature or the pump current of the slave (see Supplementary Section 2 C). The two distinct dynamical regimes in Fig. 1c are injected into the slave laser in order to analyze the synchronization quality for both high frequency chaos and slower mode-hopping dynamics. Different synchronization mechanisms emerge depending on the properties of the injected dynamics. The response of the slave laser strongly depends on the temporal and spectral structure of the injected signal, highlighting the richness of coupling phenomena in chaotic multimode systems.

The first case we study is when the master laser operates at *I*_*M*_ = 6.17 mA and exhibits a dynamics characterized by both high-frequency components and slow polarization-hopping (typically around 100 MHz). The master laser current and temperature are fixed to keep its dynamical properties constant. The slave laser current is varied between *I*_*S*_ = 2 mA and 10 mA at a fixed temperature of 20^∘^C. The slave laser modes are red-shifted due to Joule heating as its current increases [see Supplementary Section 2 C]. Furthermore, its output power increases, thereby reducing the optical injection ratio (see Supplementary Section 3 A). We study parallel injection, meaning that the *u*-polarization component of the master laser, which is its dominant polarization for this pump current, is injected into the *u*-polarization of the slave laser using a HWP. The slave laser has several PSPs in this current range, but its red polarization (*u*) remains mostly dominant (see Supplementary Fig. S2).

**Fig. 2:**
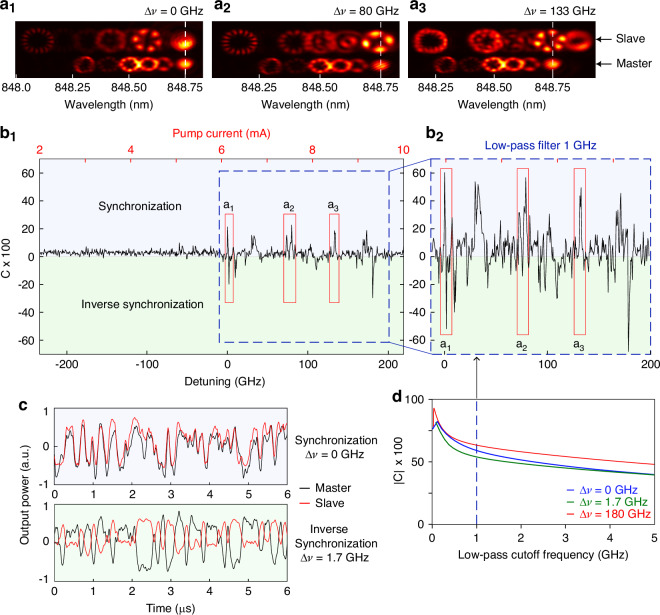
Synchronization of polarization-hopping dynamics. Spatio-spectral images in *u*-polarization of master and slave laser for Δ*ν* = 0 GHz (**a**_1_), 80 GHz (**a**_2_), and 133 GHz (**a**_3_). The camera field of view corresponds to 2600 *μm* × 740 μm in the object plane. **b**_1_ Correlation between the unfiltered and (**b**_2_) low-pass filtered (cutoff at 1 GHz) time traces in *u* − polarization of master and slave laser as a function of the detuning or pump current *I*_*S*_. **c** Low-pass filtered time traces (cutoff at 0.1 GHz) of the master (black) and slave (red) lasers for Δ*ν* = 0 GHz (top) and Δ*ν* = 1.7 GHz (bottom), corresponding to a correlation of approximately ± 80%, respectively. **d** Absolute value of correlation for Δ*ν* = 0 GHz (blue), Δ*ν* = 1.7 GHz (green), and Δ*ν* = 180 GHz (red) as a function of the low-pass filter cutoff frequency. The vertical blue dashed lines indicate the cutoff value chosen to compute the correlation coefficient shown in (**b**_2_)

Figure 2b_1_ shows the correlation between the time traces (see Methods) of *u*-polarized emissions of master and slave laser as function of the frequency detuning Δ*ν* between the two lasers, which is defined here as the frequency of mode *M*_*u*_(0, 1) minus the frequency of *S*_*u*_(0, 1) as illustrated in Fig. 2a_1_. We observe several cases of significant positive and negative values of correlation, corresponding to synchronization and inverse synchronization, respectively, which are discussed in the following.

The positive correlation peaks are related to spectral alignment between transverse modes of the master and the slave laser with high power. For Δ*ν* = 0, the spatio-spectral image in Fig. 2a_1_ shows that both the *S*_*u*_(0, 1) and the *S*_*u*_(3, 1) modes emit strongly and are spectrally aligned with the *M*_*u*_(0, 1) and *M*_*u*_(3, 1) modes, respectively. We originally expected this to yield the best synchronization since theoretically each transverse mode of the master laser would be aligned with its counterpart of the slave, but in practice perfect matching of all transverse mode frequencies is not achievable due to small differences in modal spacings between the two lasers. We also observe that for Δ*ν* = 0, when the *S*_*u*_(3, 1) mode aligns with the *M*_*u*_(3, 1) mode, its power significantly increases while neighboring modes become weaker, as discussed in Supplementary Fig. S10. This highlights the strong interaction of these two modes as their frequencies align.

Similar observations are made for the other positive correlation peaks. At Δ*ν* ≈ 30 GHz, a weak synchronization peak appears, associated with the alignment of *M*_*u*_(0, 1) with *S*_*u*_(1, 1). The peak at Δ*ν* ≈ 80 GHz corresponds to the alignment between *M*_*u*_(0, 1) and *S*_*u*_(2, 1) [see Fig. 2a_2_ and Supplementary Fig. S10]. Similarly, at Δ*ν* ≈ 133 GHz, another peak emerges from the alignment between modes *M*_*u*_(0, 1) and *S*_*u*_(3, 1) [see Fig. 2a_2_]. These examples demonstrate that synchronization can be achieved when a strong transverse mode of the master laser spectrally aligns with a transverse mode of the slave, however, it need not be the same transverse modes: spectral alignment appears to be more important than spatial alignment for successful synchronization. It should also be emphasized that the other transverse modes of the slave laser remain active and are different from those of the master laser [see Fig. 2a], meaning that synchronizing the temporal dynamics of the lasers does not require synchronizing the optical spectrum or the spatial intensity distribution.

We also observe inverse synchronization, that is negative correlation between the time traces of the *u*-polarized emission of master and slave, with the most significant examples at Δ*ν* = 1.7 GHz and at Δ*ν* = 180 GHz. Synchronization in antiphase for coupled oscillators has been known since early studies of coupled pendula and has also been observed in coupled lasers with feedback, for example in the low-frequency fluctuation regime^45^. Polarization dynamics were identified as a key factor in the emergence of inverse synchronization in refs. ^46,47^.

The first example at Δ*ν* = 1.7 GHz happens right after the alignment of the *M*_*u*_(3, 1) mode with the *S*_*u*_(3, 1) mode when the former approaches the frequency of the *S*_*v*_(3, 1) mode, as shown by the time traces of the master and slave, low-pass filtered at 0.1 GHz cutoff, in Fig. 2c. Indeed, Supplementary Fig. S8a shows that the *S*_*v*_(3, 1) mode is strongly excited at Δ*ν* = 1.7 GHz. We surmise that when injecting light into the *u*-polarization at a frequency near a *v*-polarized mode of the slave, it can create a synchronization of the *v*-polarized emission with the mode-hopping dynamics of the *u*-polarized injection signal from the master, though this is evidently not always the case. Since the emission in *u*- and *v*-polarization is highly anticorrelated in the polarization-hopping regime [see Supplementary Fig. S4], the *u*-polarized emission of the slave becomes anti-correlated to the *u*-polarized master signal since the latter is synchronized with the *v*-polarized emission of the slave. At 180 GHz detuning, we observe that the *S*_*v*_(1, 2) mode is enhanced when its frequency comes close to the *M*_*v*_(0, 1) mode.

These examples demonstrate that coupling of two multimode VCSELs can create different types of synchronization depending on system parameters, and that moreover, strong dynamic correlations can be established between transverse modes of very different order.

Finally, we analyze the master-slave correlation across different timescales by applying spectral filtering (see Methods). Figure 2d shows the evolution of the correlation as function of the low-pass filter cutoff frequency for three examples. The correlation significantly improves with correlations of up to 90% for 100 MHz cutoff at Δ*ν* = 180 GHz. Furthermore, Fig. 2b_2_ shows the correlation of the low-pass filtered time traces with 1 GHz cutoff as a function of detuning. A global increase is observed across all correlation regions. These low-frequency components represent the relatively slow polarization-hopping dynamics which appears to synchronize much better than the high-frequency components of the dynamics as demonstrated by the low-pass filtered time traces of master and slave laser at Δ*ν* = 0 and 1.7 GHz in Fig. 2c. This demonstrates that it is the polarization-hopping dynamics which is synchronized, and that one can achieve very high synchronization quality even though the BA-VCSELs are not perfectly identical.

**Fig. 3:**
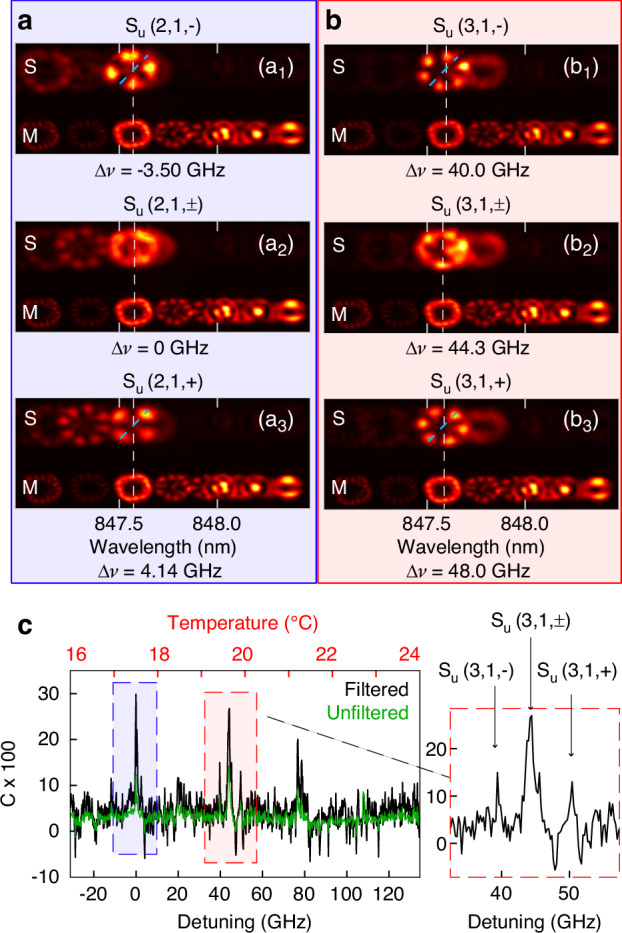
Synchronization of broadband chaotic dynamics. **a**, **b** Spatio-spectral images of *u*-polarized emission of the slave laser under injection (top) and the master laser (bottom) for *I*_*M*_ = 8.8mA and *I*_*S*_ = 4.8mA. The camera field of view corresponds to 2470 *μm* ﻿× 950 μm in the object plane. The detunings are Δ*ν* = −3.50 GHz (**a**_1_), 0 GHz (**a**_2_), 4.14 GHz (**a**_3_), 40 GHz (**b**_1_), 44.3 GHz (**b**_2_), and 48.0 GHz (**b**_3_). The symmetry axis of the transverse modes is indicated with the blue dashed lines. **c** Correlation between the *u* − polarized time traces of master and slave laser as a function of the detuning, without filter (green) and with low-pass filter at 1 GHz cutoff (black). The magnification on the right shows the three-peak scenario around Δ*ν* = 44.3 GHz

For the second case of injection that we study, the master laser is set to *I*_*M*_ = 8.8 mA and *T*_*M*_ = 20^∘^C, the slave laser is operated at a fixed current of *I*_*S*_ = 4.8 mA, and the detuning is varied via the temperature of the slave. As before, the master laser emits predominantly along the *u*-polarization, and parallel injection of the *u*-polarization is performed. These experimental parameters lead to three differences to the first case. First, the injection ratio is higher though it remains relatively weak (see Supplementary Section 3 A). Second, while the master laser has higher power, it is distributed over a larger number of transverse modes [see Fig. 3a]. Third, the master laser operates in a chaotic state with high frequency components (see Fig. 1c and Ref. ^31^) without polarization-hopping. In the following we discuss how these changes affect the synchronization.

The detuning Δ*ν* is controlled by the temperature of the slave laser, with a measured tuning coefficient of Δ*ν*/Δ*T* = 20.7GHz/K. We define here that Δ*ν* = 0 GHz when the mode *S*_*u*_(2, 1) is aligned with the mode *M*_*u*_(6, 1), which is the dominant mode of the master and plays an important role in the following. The spatio-spectral images in Fig. 3 reveal that this mode lases in both orientations, *M*_*u*_(6, 1, + ) and *M*_*u*_(6, 1, − ), at the same time, and with a negligible splitting Δ*ν*_*O*_. This property of the master strongly influences the synchronization behavior.

Figure 3c shows several regions of positive correlation. The region around Δ*ν* ≈ 0 GHz stems from synchronization of the *M*_*u*_(6, 1, ± ) modes with the *S*_*u*_(2, 1, ± ) modes, which are very weak in free-running operation but can be strongly excited by the injection. A closer look shows that there are actually three successive correlation peaks: *S*_*u*_(2, 1, − ) is excited at Δ*ν* = − 3.5 GHz, both *S*_*u*_(2, 1, + ) and *S*_*u*_(2, 1, − ) are excited at Δ*ν* = 0 GHz, and *S*_*u*_(2, 1, + ) is excited at Δ*ν* = 4.14 GHz as shown in Fig. 3a. We attribute this behavior to the splitting between the *S*_*u*_(2, 1, − ) and *S*_*u*_(2, 1, + ) modes, which seems to be about Δ*ν*_*O*_ ≈ 7.5 GHz (see also Supplementary Section 2 A d): these two modes are excited alone when the *M*_*u*_(6, 1, ± ) modes aligns spectrally with them, and their superposition is created when *M*_*u*_(6, 1, ± ) is in between the two orientations of *S*_*u*_(2, 1). The same behavior is found when the *M*_*u*_(6, 1, ± ) modes come close to the *S*_*u*_(3, 1, ± ) modes [see Fig. 3b and the magnification in Fig. 3c]. We associate this scenario to the *M*_*u*_(6, 1, ± ) modes lasing simultaneously, which creates an azimuthally uniform intensity distribution that is able to excite both orientations of the slave laser modes equally well.

However, spectral alignment alone does not guarantee strong synchronization. For instance, around Δ*ν* ≈ 115 GHz only a low correlation around 9% is observed despite the spectral matching between the *M*_*u*_(6, 1, ± ) modes and the *S*_*u*_(4, 1) mode. The third region of positive correlation shows a somewhat different scenario: at Δ*ν* ≈ 76 GHz, the *M*_*u*_(5, 1) mode of the master, which lases only in a single orientation, excites a superposition of the *S*_*u*_(2, 1, + ) and *S*_*u*_(2, 1, − ) modes. However, the correlation is low, probably because the *M*_*u*_(5, 1) has less power than the *M*_*u*_(6, 1, ± ) modes.

This second injection experiment confirms our earlier observation that spectral alignment between a strong mode of the master and a transverse mode of the slave laser is an important prerequisite for synchronization. While the transverse modes of the master and slave lasers that are coupled can be different, the spatial structure of the master laser mode can also play an important role: simultaneous lasing of the master laser mode in both orientations favors the emergence of three correlation peaks, in which different orientations of the same transverse mode of the slave laser are excited. Furthermore, we find that a higher injection ratio does not necessarily lead to higher correlations, which may be due to the master power being spread across more modes, while power concentration in a single mode seems to play a key role.

Applying a low-pass filter improves the correlations to some extent, typically reaching peak values between 20% and 30% with cutoff frequencies around 0.5 to 1 GHz [see Fig. 3c], but these values are below those found for the first case [see Fig. 2]. We believe this is related to the type the master laser dynamics, which is chaotic with high frequency components up to 20 GHz [see Fig. 1c], in contrast to the slower polarization-hopping dynamics in the first case. Whereas the slow polarization-hopping dynamics creates strong synchronization which is revealed by low-pass filtering, the chaotic dynamics in the second case relies on high-frequency components, so filtering does not help much. It seems that fast chaotic dynamics is harder to synchronize. The absence of inverse synchronization in the second case is explained by the absence of polarization-hopping dynamics to which we attribute the inverse synchronization in the first case. In summary, the second injection experiment confirms the importance of spectral alignment for synchronization [see also Supplementary Fig. S9]. However, we also find significant differences between the two cases, demonstrating the diversity of synchronization scenarios and their dependency on the dynamical and spatial properties of the master laser.

## Discussion

Our experiment constitutes a lab-scale demonstration of synchronization between systems with complex spatio-temporal dynamics. By exploiting the intrinsic and controllable chaotic dynamics of broad-area VCSELs, it also constitutes the simplest and most practical architecture so far for multiple-user physical-layer secure communication schemes at high bit rates. Such systems would benefit from the highly complex and fast fluctuations of the laser dynamics and the spatial aspect of the dynamics involving a large number of laser modes. Moreover, the high-dimensional spatio-temporal dynamics of broad-area VCSELs could also be exploited in future photonic reservoir-computing architectures^48^.

Owing to the multimode nature and intrinsic complexity of the VCSELs, the dynamics of the coupled system is very rich, and we observe different scenarios including synchronization of ultrafast chaos, polarization-hopping dynamics, and inverse synchronization. The measured correlations are generally low of the order of 20%, which could be due to the very low transmission through the top mirror of the VCSELs, mismatch in the device structures and the intrinsic mode competition. However, correlations up to 90% are observed for low-pass filtered polarization-hopping dynamics. Synchronization is typically observed when one of the dominant modes of the master laser is spectrally aligned with a mode of the slave laser, but does not require a careful matching of their spatial profiles. This flexibility and the multitude of possible synchronization scenarios give hope that further improvements of the synchronization quality are possible. Still, the high correlation of the low-pass filtered polarization-hopping dynamics is promising for applications such as private key sharing^49,50^ that typically require both high-dimensional chaos and good synchronization quality, but not very high speeds.

Our work opens new perspectives concerning the synchronization of complex lasers and beyond. We show that synchronization of the temporal dynamics does not require or imply that other system properties like the spectrum and the spatial profiles become identical as well. Hence, a more precise exploration and classification of synchronization scenarios for coupled spatio-temporal systems will be needed, with implications in other scientific fields. Beyond the temporal dynamics, further investigations of the spatial dimension appear promising, for example by using different cavity shapes^51–53^ or shaping the injected beam. Tuning the birefringence or introducing controlled anisotropies could help to isolate polarization states and their associated spatial modes. By systematically varying the number and nature of the excited modes, one could assess how the modal complexity influences the robustness, speed, or nature of synchronization. Such control would allow for a more precise exploration of the transition from low-dimensional to high-dimensional chaotic synchronization in spatially extended laser systems.

## Materials and methods

### Correlation measurement

The synchronization quality between the master and the slave lasers is calculated using the well-known correlation coefficient *C*(Δ*t*)

1 $$C(\Delta t)=\frac{\langle [{P}_{M}(t)-\langle {P}_{M}\rangle ][{P}_{S}(t+\Delta t)-\langle {P}_{S}\rangle ]\rangle }{\sqrt{\langle {[{P}_{M}(t)-\langle {P}_{M}\rangle ]}^{2}\rangle \langle {[{P}_{S}(t)-\langle {P}_{S}\rangle ]}^{2}\rangle }}$$

where *P*_*M*_(*t*) and *P*_*S*_(*t*) are the output powers (i.e., time traces) of the master and slave lasers, respectively, that are measured with the high-speed photodetectors. The time average is denoted by 〈〉, and Δ*t* is a time shift. The correlation is computed as function of Δ*t* for each detuning Δ*ν*. Figures 2b and 3c, however, only show the correlation at the Δ*t* for which it is maximal. This time shift of Δ*t* ≈ 3.78 ns corresponds to the path-length difference between the master and slave signals, and the maximum of the correlation is indeed obtained at this delay, confirming that zero-lag synchronization is computed and observed. For detunings where the master and slave lasers are not synchronized, the correlation is theoretically expected to vanish. However, a persistent residual correlation remains observable in our system, fluctuating around *C* = 3% for the unfiltered data. This non-vanishing baseline arises from the reflection of the master laser from the top mirror of the slave laser. We verified that even with the slave laser switched off, a small fraction of the master signal is reflected back, producing a weak but measurable correlation. This weak correlation also appears at a time lag Δ*t* corresponding to the propagation delay along the signal pathways.

### Filtering of time traces

In addition to analyzing the correlation of the original measured time traces, we also investigated the effect of spectral filtering on the correlation. A Butterworth low-pass filter was chosen due to its simplicity of implementation and its flat frequency response in the passband, which ensures that the preserved frequency components are not distorted by filtering artifacts. Different cutoff frequencies *f*_*c*_ were tested, ranging from the full bandwidth of the measured signal (23 GHz) down to 10 MHz, allowing us to investigate how the correlation evolves across different frequency ranges.

The transfer function of an *n*th-order Butterworth filter as function of RF-frequency *f* is given by

2 $$H(f)=\frac{1}{\sqrt{1+{(f/{f}_{c})}^{2n}}}$$

where *f*_*c*_ is the cutoff frequency. It corresponds to the point where the transfer function drops to -3 dB, marking the boundary between the passband and the stopband. In our case, a filter of order *n* = 2 was used, providing a suitable trade-off between high-frequency attenuation and filter stability. We verified that the type and order of the low-pass filter (Butterworth, zero-phase, or linear-phase FIR) do not affect the synchronization coefficient, indicating that filtering does not distort the phase or delay in a way that impacts our results.

## Supplementary information

Supplementary Information for “Synchronization of complex spatio-temporal dynamics with lasers”

## Data Availability

Data underlying the results presented in this paper are not publicly available at this time but may be obtained from the authors upon reasonable request.
