# Supplementary material for: Synchronization of complex spatio-temporal dynamics with lasers
Source: Light Sci Appl. 2026 Feb 28;15:131. doi: 10.1038/s41377-026-02198-5 (PMC12949983; doi:10.1038/s41377-026-02198-5)
Supplement: Supplementary file 1 — Supplementary Information for “Synchronization of complex spatio-temporal dynamics with lasers” [file 41377_2026_2198_MOESM1_ESM.pdf]

Supplementary Information for "Synchronization of complex spatio-temporal dynamics with lasers"

Jules Mercadier[*,1,2], Stefan Bittner[1,2] and Marc Sciamanna[*,1,2]
[1]Université de Lorraine, CentraleSupélec, LMOPS F-57000 Metz, France
[2]Chaire Photonique, LMOPS, CentraleSupélec, 57070 Metz, France
* Corresponding authors : jules.mercadier@centralesupelec.fr; marc.sciamanna@centralesupelec.fr

Supplementary Section 1: Experimental setup

The experimental setup is presented in Fig. S1 in more detail. The lasers under study are broad-area VCSELs (Frankfurt Laser Company FL85-F1-P1N-AC) with a circular output aperture of 15 μm diameter, electrically pumped through a ring contact. The output beam of the master laser is collimated by a 40× microscope objective (Obj1, NA = 0.6). An optical isolator (OI) is placed immediately after the master laser to prevent any undesirable optical feedback that could influence its intrinsic dynamics. A beam splitter (reflexion: 70 / transmission: 30) directs part of the master laser signal to the detection arm (30%), and part of the signal (70%) is injected into the slave laser. In the same way, part of the slave laser signal is collimated and sent to the detection arm.

The detection arm allows to measure the temporal dynamics, the optical spectra, and spatio-spectral images of the two lasers. Two fiber couplers with 20× objectives (Obj2, NA = 0.5) are employed to couple the beams into multimode graded-index fibers (Thorlabs M116L02, 50 μm core diameter) connected to high-speed photodetectors (Newport 1484-A-50, 22 GHz bandwidth). The RF-signals from the photodetectors are amplified by RF-amplifiers (SHF S126A) yielding a gain of 29 dB over a frequency range spanning from 80 kHz to 25 GHz, and measured by an oscilloscope (Tektronix DPO72340SX, 23 GHz bandwidth). Time traces were recorded with a sampling rate of 50 GS/s, using a record length of 10^6 points (corresponding to a total acquisition time of 20 μs). Because the detection chain is AC-coupled, the DC component of the optical signal is removed. The measured voltage therefore reflects only the temporal fluctuations of the laser's optical power, excluding its constant (DC) offset. Before computing the correlation, the mean value of each time trace was subtracted. No additional detrending was applied. The normalization of the variance is inherently included in the definition of the correlation coefficient. Alternatively, the multimode fibers can be connected to an optical spectrum analyzer (OSA, Anritsu MS9740A, 30 pm resolution) to examine the spectral composition of the laser emission. Optical isolators are used to prevent reflections from the fiber facets, and the entrance polarizers of the isolators are aligned to transmit only the u-polarization of the lasers (unless otherwise noted).

By means of several lenses (L2, 100 mm focal length) and beam splitters, the output facets of the two BA-VCSELs are imaged onto the entrance slit of an imaging spectrometer (Princeton Instruments SpectraPro HRS-500), with a spectral resolution of 30 pm and 1800 g/mm grating. Due to the different beam paths and lens configurations used, the magnification is different for each laser. The slit of the spectrometer is fully open so that the camera in the focal plane of the spectrometer measures the spectrally-resolved near-field intensity distributions of the two VCSELs, that is, the intensity patterns of the different transverse modes are dispersed horizontally as function of their wavelength. The images of the two VCSELs are offset vertically, and a beam displacer (Thorlabs BD27, 2.7 mm beam separation) preceded by a half-wave plate is used to vertically separate the u- and v-polarizations of the slave laser as well. Thus the two polarizations of the slave laser and the u-polarization of the master laser are measured in parallel.

The signal of the master laser passes through the collimation objective (Obj1, NA = 0.6), a lens (L1, 200 mm focal length), another lens of the same type and finally the objective (Obj1) in front of the slaver laser. The lenses are aligned such that the output facet of the master laser is imaged onto the output facet of the slave laser with 1x magnification. Furthermore, additional cameras in the far-field planes of the lasers (not shown in Fig. S1) are used to ensure that the injected beam is parallel to the output beam of the slave laser. The optical isolator after the master laser is used to select only its u-polarization for transmission to the slave laser, and a half-wave plate is used to align the polarization of the master signal with the u-polarization axis of the slave laser for parallel optical injection. A variable neutral density filter allows to change the power of the injected signal. The results presented in this article were obtained for minimal attenuation (maximal injection power).

As previously discussed, although the injection ratio is difficult to estimate precisely in practice, it remains very low and does not appear to directly affect the observed correlation levels. Another crucial factor is the optical alignment, which is essential to ensure optimal coupling between the two lasers. In addition to aligning the u-polarizations to allow for parallel injection, the angle at which the master beam enters the slave laser cavity had to be finely adjusted. To verify and optimize this alignment, both near-field and far-field profiles of the master

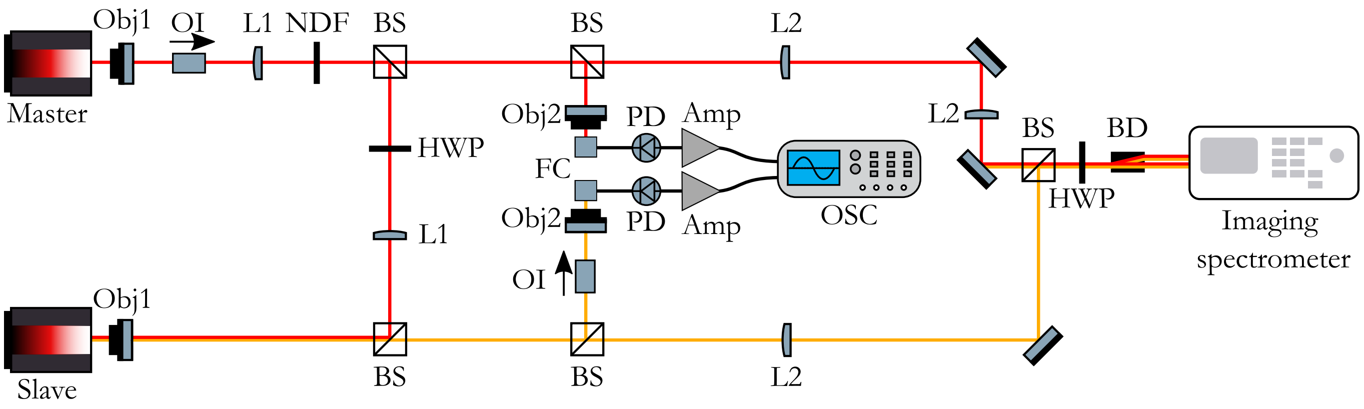

FIG. S1. Experimental setup. Obj1: 40x objective (NA = 0.6); Obj2: 20x objective (NA = 0.5); OI: optical isolator; NDF: neutral density filter; BS: beam splitter; HWP: half-wave plate; FC: fiber coupler; PD: Photodetector; Amp: RF-Amplifier; OSC: oscilloscope; L1 (L2): lens with 200 mm (100 mm) focal length; BD: beam displacer.

and slave lasers were compared and carefully overlapped. This alignment proved to be crucial and technically challenging to implement.

Supplementary Section 2: Properties of free-running lasers

A. Polarization and spectrum

a. LI-curves and polarization-switching points

To identify the principal polarization axes of the BA-VCSELs used in this work, a polarizer in a motorized rotation stage was employed to measure the optical power transmitted through the polarizer as function of its orientation for both lasers. Using a power meter, measurements were taken every 10° over the full current range of 2 to 10 mA as shown in Fig. S2. The resulting profiles exhibit two distinct extrema in emitted power at 0° (or equivalently 180°) and 90°, revealing the two orthogonal polarization axes. Depending on the pump current and the temperature of the laser, the dominant polarization switches between these two orientations at specific PSPs, while the u- and v-polarization axes themselves remain fixed. In most cases, the 90° axis (referred to as u-polarization) dominates, while the 0° / 180° axis is referred to as v-polarization. See Ref. [S1] for more details. It should be noted that we define the polarizer angle with respect to the polarization axes of the VCSELs as a convenient reference frame to facilitate visualization and comparison. These axes generally do not coincide with the horizontal and vertical axes of the laboratory frame.

b. Optical spectrum and birefringence

Measurements of the optical spectra provide important information on the properties of the lasers under study.

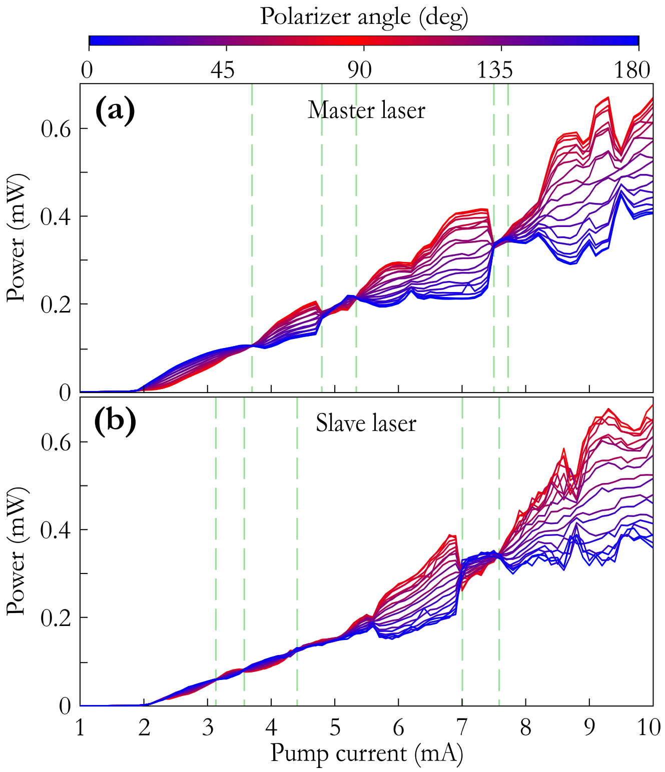

FIG. S2. LI-curves of master (a) and slave laser (b) for 19 polarizer angles ranging from 0° to 180°. The color corresponds to the polarizer angle, where u-polarization (at 90°) is shown in red and v-polarization (at 0° and 180°) in blue. The dashed green lines indicate the polarization switching points (PSPs).

First, the wavelengths of the individual modes are clearly identified, with the fundamental mode appearing at the longest wavelength (right side of the spectrum), and higher-order transverse modes at shorter wavelengths (as discussed in more detail in Ref. [S1]). Figure S3 shows the optical spectra of the master laser measured along

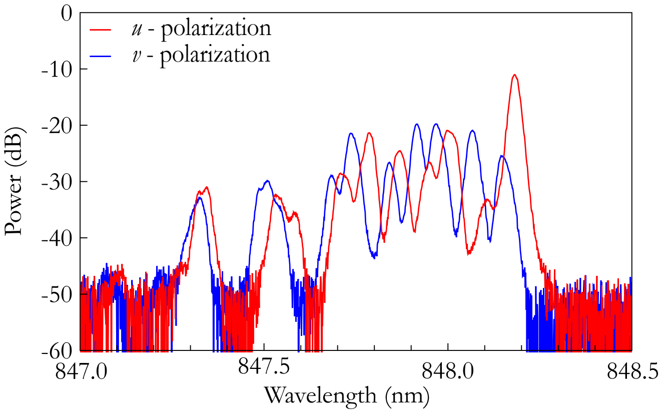

FIG. S3. Optical spectra of the master laser at a pump current of $I_M = 6.5$ mA and a temperature of $T_M = 20°$C, measured along the u- (red) and v-polarization (blue) axes.

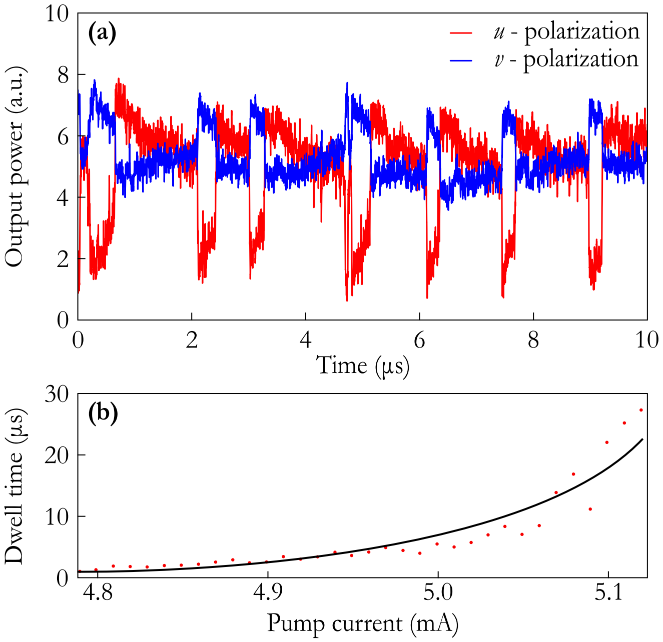

FIG. S4. (a) Time traces of the master laser at I_M= 4.95 mA, low-pass filtered at a cutoff of 0.1 GHz, for the two orthogonal polarization axes u (red) and v (blue). (b) Mean dwell time of the time traces in one polarization state as function of the pump current.

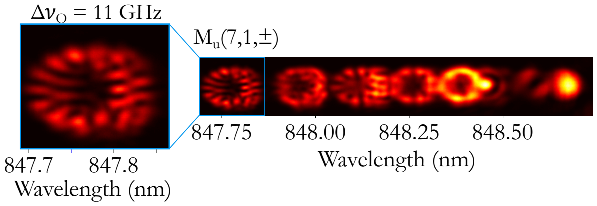

FIG. S5. Spatio-spectral image of the free-running master laser in $u-$polarization for I_M= 9.8 mA with the modes $M_u(7,1,+)$ and $M_u(7,1,-)$ highlighted in the inset in blue. They are offset laterally due othe the non-vanishing orientation splitting $\Delta\nu_O \approx 11$ GHz.

the two dominant polarization axes, u and v, for a fixed pump current of $I_M = 6.5$ mA. This measurement reveals that the optical power is distributed differently among the transverse modes depending on the polarization, and also highlights a spectral shift between the two polarization components. We assign the label "red" ("blue") to the u- (v-) polarization whose spectrum is at longer (shorter) wavelengths.

This spectral separation is due to the intrinsic birefringence of the VCSEL which plays a critical role in polarization dynamics. We experimentally measured the birefringence frequency from the spectral separation between the same transverse modes in the two orthogonal polarizations. Although the birefringence may vary slightly depending on the mode, this analysis allowed us to estimate $\Delta\nu_b = 8.8$ GHz for the master laser and $\Delta\nu_b = 8.4$ GHz for the slave laser. We explicitly chose two VCSELs with very similar birefringence and close central wavelength value.

c. *Polarization-hopping dynamics*

The VCSELs under study exhibit several polarization switching points (PSPs) at which the dominant polarization changes (see Fig. S2), such as in the case of the master laser at 3.6 mA, as well as points at which the power in the two polarization states becomes almost equal without switching, such as the master laser at 6.2 mA. These points are sometimes accompanied by a dynamical regime known as polarization hopping. As illustrated in Fig. S4(a), this regime is characterized by intensity fluctuations in one polarization (u), with an anti-correlated response in the orthogonal polarization (v). Due to the relatively long timescales of this dynamics, the associated intensity fluctuations contribute significantly to the low-frequency components of the RF spectrum, predominantly below 500 MHz.

This behavior has been reported in various VCSEL systems [S2-S4], and the spontaneous polarization switching can be driven by noise or by deterministic nonlinear dynamics. The temporal behavior within this regime is characterized by the dwell time, defined as the average duration the system remains in one polarization state before switching. Figure S4(b) presents the measured dwell time as a function of pump current, showing an exponential increase with increasing current. This trend is characteristic of deterministic polarization chaos [S4].

d. *Orientation of transverse modes*

Due to the circular geometry of the VCSEL cavity, all transverse modes (m, n) with $m > 0$ exist in two different orientations, designated by + (−) if they are (anti-)

symmetric with respect to a chosen axis. In an ideally symmetric cavity, the modes with + and − orientations are degenerate.

Figure 1(b_2) presents polarization-resolved spatio-spectral measurements of the master laser under free-running conditions for I_M= 6.5 mA. Several key observation can be made: first, we typically observe modes with the same transverse pattern in both polarization states, however, there is a clear spectral shift between them due to the birefringence as discussed above. Second, the intensities in the two polarizations are generally not the same (see Fig. S3). Third, for a given (m, n), the u- and v-polarizations usually exhibit different transverse orientations, with a − symmetry in u-polarization corresponding to a + symmetry in v-polarization, which is attributed to gain competition [S5,S6]. In short, usually each transverse mode lases in both polarizations but with different spatial orientations and different intensities. However, in some cases, such as mode $M_u(6, 1)$ in Fig. 3, the measured spatial pattern shows that both orientations lase in the same polarization state (under free-running conditions).

Figure S5 shows the spatio-spectral image of the master laser at 9.8 mA. In this example, the mode (7, 1) lases in both orientations in u-polarization, however, we see that the $M_u(7, 1, +)$ and the $M_u(7, 1, −)$ mode are not degenerate as expected for a perfectly symmetric system, but spectrally shifted. We designate this frequency separation between the + and − orientations of a given transverse mode within the same polarization as $\Delta\nu_O$. This splitting is usually below the resolution of standard optical spectrum analyzers or imaging spectrometers, making it difficult to measure directly. In the case of Fig. S5, it is quite large with $\Delta\nu_O \approx 11$ GHz. In other instances, like the mode $M_u(6, 1)$ in Fig. 3, it is close to 0.

Moreover, the progressive excitation of different orientations under optical injection, such as the modes $S_u(2, 1)$ and $S_u(3, 1)$ previously presented in Fig. 3, enables an indirect estimation of $\Delta\nu_O$ around 7.64 GHz and 8 GHz, respectively, by considering the secondary correlation peaks observed near the principal one. Overall, the degeneracy splitting $\Delta\nu_O$ is strongly mode-dependent and may range from nearly 0 up to at least 11 GHz, highlighting the complexity of polarization and orientation resolved transverse mode spectra in BA-VCSELs.

B. Comparison of master and slave laser

Among a batch of five nominally identical BA-VCSELs from the same manufacturer, a detailed characterization was performed on each device to identify the most suitable pair for the injection experiment. The selection was guided by two key criteria: similarity in birefringence frequency $\Delta\nu_b$ and in wavelength of the lasing modes. As the laser dynamics are strongly influenced by birefringence, we selected two devices exhibiting closely matched values, $\Delta\nu_b = 8.8$ GHz for the master laser and

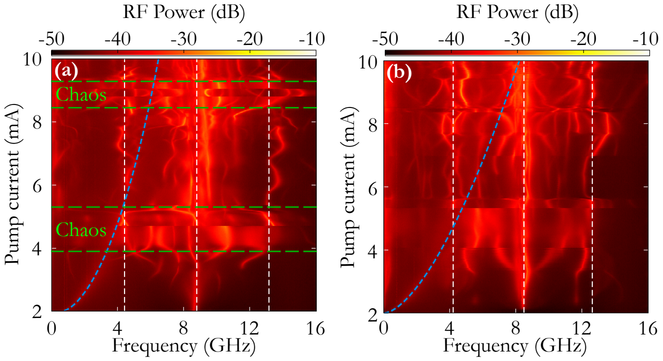

FIG. S6. RF-spectra of the master (a) and slave laser (b) as a function of the pump current from 2 to 10 mA. The RF-spectra were measured for a polarizer angle of 45°. The RF-spectra are smoothened by a sliding average over 10 points (0.5 MHz). The birefringence frequencies $\Delta\nu_b$ as well as $0.5\Delta\nu_b$ and $1.5\Delta\nu_b$ are indicated with white dashed lines, and the relaxation oscillation frequency with blue dashed lines. The green dashed lines in (a) delimit the current regimes in which the master lasers exhibits high-frequency chaotic dynamics.

$\Delta\nu_b = 8.4$ GHz for the slave. Spectral alignment was also critical, since frequency detuning strongly affects the injection dynamics. Both lasers exhibited comparable emission wavelengths over the considered current range (2–10 mA), ranging from 847.6 to 848.6 nm at a common temperature of 20°C.

Prior to the injection experiment, the intrinsic free-running behavior of each laser was analyzed. Figure S2 presents polarization-resolved LI curves for both devices. While both exhibit well-defined orthogonal polarization axes, their PSPs differs significantly, even though the u-polarization (red) generally dominates, which is the polarization axis we chose for injection. Further insights were obtained from the analysis of the RF-spectra shown in Fig. S6, which displays the evolution of RF-spectra as function of the pump current measured along the 45° polarization axis, that is, u- and v-polarization are measured with equal efficiency on the photodetector. Consistent with previous studies [S7], the dominant frequency components appear around the birefringence frequency $\Delta\nu_b$, as well as its subharmonic and harmonic ($0.5\Delta\nu_b$ and $1.5\Delta\nu_b$, white dashed lines). Relaxation oscillations are also visible (blue dashed lines). The bifurcations, often coinciding with PSPs, are clearly reflected in changes of the spectral content. Some bifurcations mark the begin of a regime of high-frequency chaos, delimited by the green dashed lines in Fig. S6(a) for the master laser. The chaotic regions were identified using the noise titration method as well as the Grassberger-Procaccia algorithm [S7].

These analyses show that although the two selected VCSELs share key parameters such as birefringence and emission wavelength, their intrinsic dynamics are far from being stationary and furthermore differ significantly. This makes polarization-resolved injection exper-

iments between them a challenging task. Once the pair of VCSELs was identified, polarization alignment in the injection path was ensured using a half-wave plate (see Fig. S1). Due to intrinsic birefringence and structural anisotropies, the u- and v-polarization axes of each VCSEL differ: for the master, $u = 160°$ and $v = 70°$; for the slave, $u = 150°$ and $v = 60°$ (note that these angles are given with respect to the horizontal axis of the laboratory frame, in contrast to the convention established in section 2 A). Combined with the 45° rotation introduced by the optical isolator and further rotations from subsequent reflections, proper alignment of the injected field was achieved by tuning the HWP to ensure that the u-polarization of the master was injected into the u-axis of the slave to achieve polarization matching.

Furthermore, the estimated relaxation oscillation frequencies range from 0.7 to 6.7 GHz for the master laser and from 0.6 to 7.9 GHz for the slave laser as a function of pump current, but no significant influence of their matching on the synchronization quality is observed in our experiments.

C. Dependency on laser parameters

The emission properties of the BA-VCSELs exhibit a strong dependency on both pump current and temperature. As expected for semiconductor lasers, increasing the pump current induces a red-shift of the emission wavelength due to Joule heating. In addition, the transverse mode structure evolves with the current, often accompanied by polarization-dependent redistribution of intensity among the modes. For instance, higher-order modes emerge at increased current levels, with distinct spectral positions and polarization preferences. Thermal effects further shift the emission spectrum. A linear redshift is observed as a function of temperature and pump current, with typical tuning coefficients of approximately 0.05 nm/°C and 0.136 nm/mA. These shifts impact the spatial overlap and the spectral alignment between the two VCSELs. Accurate current and temperature control is therefore critical to maintain spectral alignment, especially in weak injection regimes.

Supplementary Section 3: Injection experiments

A. Injection ratio

The injection ratio is a fundamental parameter for coupling two laser systems. In this study, it is quantified as the injection strength κ_{inj}, calculated as the power from the master laser in the u-polarization at the input of the slave laser divided by the total output power of the slave laser $P_{\text{S,fr}}$,

$$\kappa_{\text{inj}} = \frac{P_{\text{inj}}}{P_{\text{S,fr}}} \tag{S1}$$

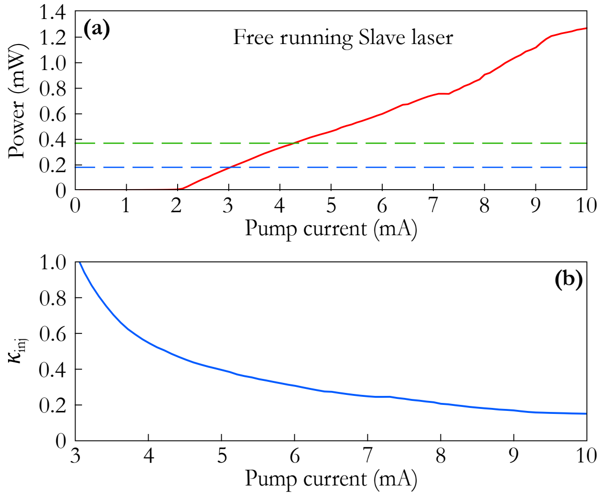

FIG. S7. (a) Total output power $P_{\text{S,fr}}$ of the slave laser in free-running conditions as a function of the pump current. The green (blue) dashed lines represent the injected power from the master laser for $I_M = 8.8$ mA ($I_M = 6.17$ mA), respectively. (b) Corresponding injection strength κ_{inj} between the master and slave lasers for $I_M = 6.17$ mA, plotted as a function of the slave laser's pump current I_S.

The measured injection power accounts for optical losses due to the isolator, beam splitter, and other optical elements. Furthermore, the injection strength can be reduced via a variable neutral density filter (see Fig. S1), but it was set to minimal attenuation in the experiments presented here. $P_{\text{S,fr}}$ is the free-running total output power of the slave laser, measured directly at the output, and includes both polarization components.

In the first case of injection studied in the paper, the master laser operates at fixed parameters ($I_M = 6.17$ mA) and delivers an input power of approximately $P_{\text{inj}} = 0.18$ mW at the entrance of the slave laser, indicated by the blue dashed line in Fig. S7(a). The output power of the slave $P_{\text{S,fr}}$ varies nearly linearly with pump current as shown in Fig. S7, and this variation determines the resulting injection strength κ_{inj}, plotted in Fig. S7(b). It is important to note that these values do not take into account reflections at the slave input facet or other factors affecting the coupling efficiency; they only reflect the available optical power at that point.

As the slave pump current I_S increases, its output power increases, leading to a decrease in κ_{inj}. For example, for the correlation peaks labeled a_1, a_2, a_3, and the final peak shown in Fig. 2, corresponding to slave currents of approximately 6.0, 7.5, 8.5, and 9.5 mA, respectively, the injection strengths are estimated around 0.30, 0.22, 0.17, and 0.14. This represents a reduction by a factor of almost two of the injection strength between the first and last peak. However, the correlation values are roughly the same in all four cases, suggesting that moderate variation in injection strength does not significantly impact correlations in this regime.

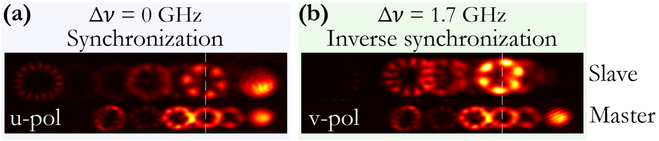

FIG. S8. Spatio-spectral image of the master laser in $u-$polarization for I_M= 6.17 mA (bottom), with the spatio-spectral image of the slave laser at $\Delta\nu = 0$ in $u-$ polarization (a) and at $\Delta\nu = 1.7$ GHz in $v-$polarization (b).

In the second case of injection we study, at I_M = 8.8 mA and I_S = 4.8 mA, the injected power reaching the slave is approximately 0.37 mW [green dashed line in Fig. S7(a)], while the total output power of the slave laser is 0.44 mW, corresponding to an injection strength of $\kappa_{\text{inj}} = 0.84$.

However, it is important to note that the values of κ_{inj} presented here should be interpreted with caution. The measured master laser power corresponds to the value at the front of the slave laser. It is not possible to directly determine the portion that actually couples into the slave cavity. A rough estimate can nevertheless be made. In fact, assuming a typical front-facet reflectivity of $> 90\%$, only a fraction of less than 10% of the master power effectively enters the cavity. Similarly, the total output power of the slave laser used here does not reflect the real intracavity power, and is therefore underestimated. So while the injection strength is rather weak in our experiment, it allows us to rule out trivial synchronization effects driven purely by high-power injection.

B. Inverse synchronization

The inverse synchronization phenomenon observed in the first injection case has been attributed to polarization-related dynamics. Figure S8 considers the first synchronization region and shows in panel (a) the case of $\Delta\nu = 0$, where the $S_u(3,1)$ mode is spectrally aligned with the $M_u(3,1)$ mode. As a result, its intensity increases significantly, while neighboring modes are suppressed. This spectral alignment illustrates the strong mode interaction when their frequencies coincide. Figure S8(b) shows a similar effect, but in the orthogonal polarization of the slave laser (v-axis) at $\Delta\nu = 1.7$ GHz: the $S_v(3,1)$ mode aligns with the $M_u(3,1)$ mode, leading to an intensity increase of $S_v(3,1)$ at the expense of adjacent modes. This alignment coincides with the occurrence of the negative correlation peak, suggesting anti-correlation between the u-polarized emission of the master and slave laser appears because the v-polarized emission of the slave is correlated with the master signal. This example illustrates that the excitation of a slave laser mode in the v-polarization systematically coincides with the occurrence of the inverse synchronization peaks.

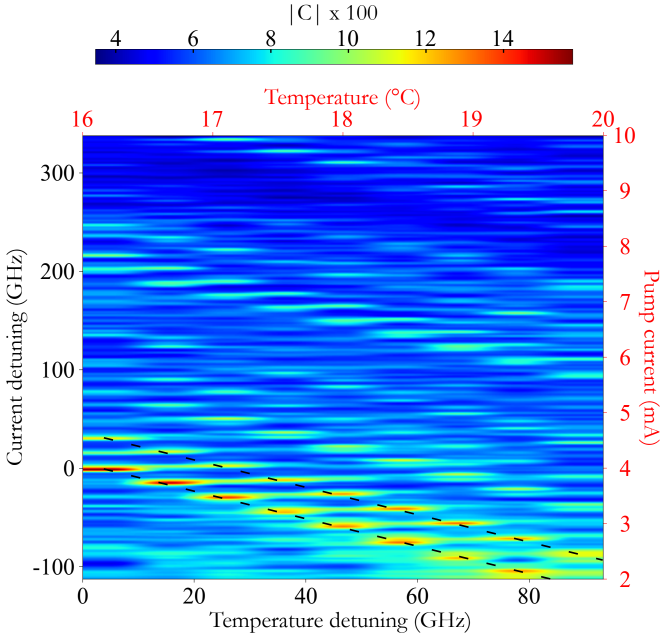

FIG. S9. Correlation map between the master laser operated at T_M = 19°C and I_M = 9 mA in the u-polarization, and the slave laser as a function of its temperature (horizontal axis) and injection current (vertical axis). The color scale represents the absolute value of correlation ($|C| \times 100$) of the unfiltered time traces. The black dashed lines follow the two principal series of correlation peaks.

C. Injection with variation of slave current and temperature

Here, we present the evolution of the correlation between the master and slave lasers as a function of several experimentally scanned parameters. The master laser is held fixed at I_M = 9 mA and T_M = 19°C, while the slave laser is scanned in both temperature (0.5° step) and injection current (0.01 mA step). The time traces are measured as function of the pump current, following the same procedure used in the first injection case discussed earlier, while keeping the slave temperature fixed. Then the slave temperature is increased followed by another sweep of the current, covering a range of 16°C to 20°C.

Figure S9 displays the absolute value of the correlation of the unfiltered time traces as function of slave temperature and current. The temperature and current shifts are converted to the corresponding detuning, and the total detuning $\Delta\nu$ is the sum of the current and temperature detunings. The map reveals several series of correlation peaks, with the first and strongest one emerging at $\Delta\nu = 0$ GHz. These correlation peak series appear as diagonals, reflecting conditions of spectral alignment between specific transverse modes of the master and slave lasers. The key implication of these diagonal series is that spectral alignment is a necessary condition for correlation, and can be achieved with both current and tem-

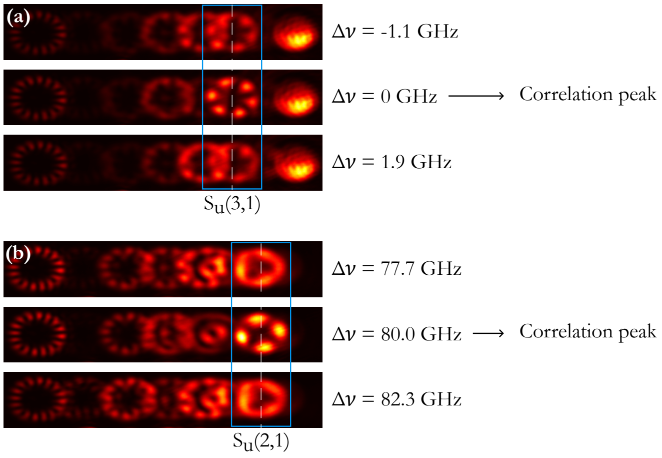

FIG. S10. Spatio-spectral images of the injected slave laser in u-polarization for different values of the detuning $\Delta\nu$ for the first injection case with $I_M = 6.17\,\text{mA}$. (a) At $\Delta\nu = 0\,\text{GHz}$ we find the spectral alignment of the slave mode $S_u(3,1)$ with the master mode $M_u(3,1)$, (b) and at $\Delta\nu = 80\,\text{GHz}$ the alignment of $S_u(2,1)$ with $M_u(0,1)$. The spectrally aligned slave modes are highlighted by the blue rectangles.

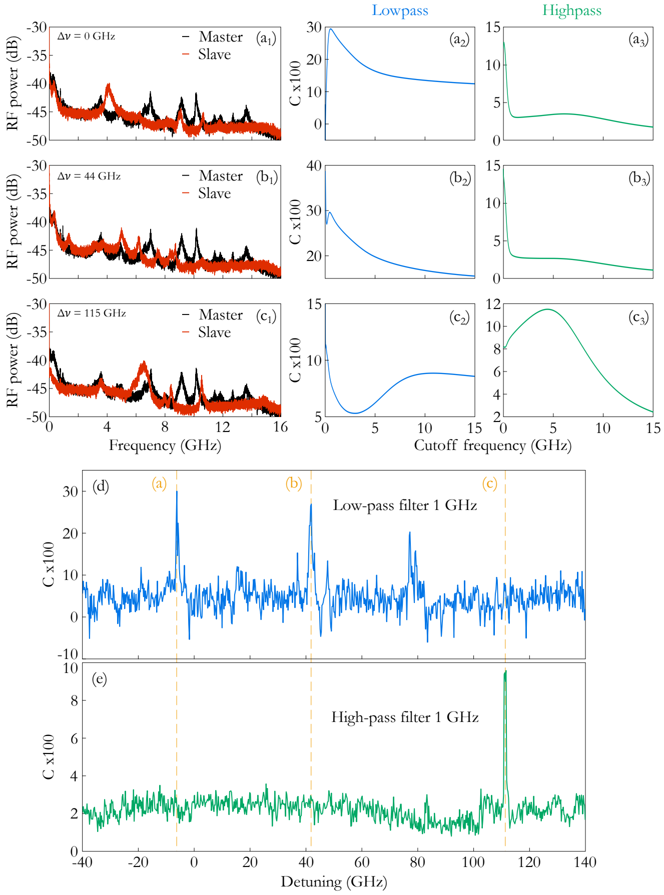

FIG. S11. (a,b,c$_1$) RF-spectra of the master (black) and slave (red) lasers. Evolution of the correlation as a function of cutoff frequency for low-pass (a,b,c$_2$) and high-pass (a,b,c$_3$) filtering for three detuning values: (a) $\Delta\nu = 0\,\text{GHz}$, (b) $\Delta\nu = 44\,\text{GHz}$, and (c) $\Delta\nu = 115\,\text{GHz}$. (d) Correlation as a function of detuning using a low-pass filter at 1 GHz cutoff and (e) a high-pass filter at 1 GHz.

perature variation. However, we also observe that the correlation varies along these diagonals, suggesting that other experimental parameters also influence the quality of synchronization.

D. Enhancement of Slave laser mode

The synchronization regions observed in the first injection case ($I_M = 6.17\,\text{mA}$) are closely associated with spectral alignment between a strong transverse mode of the master laser and a transverse mode of the slave laser as function of the frequency detuning $\Delta\nu$, see Fig. 2. Several distinct correlation peaks were identified, notably at $\Delta\nu = 0\,\text{GHz}$ and $\Delta\nu = 80\,\text{GHz}$, each corresponding to the alignment of specific lasing modes. When the slave mode $S_u(3,1)$ becomes spectrally aligned with the master mode $M_u(3,1)$ at $\Delta\nu = 0\,\text{GHz}$, the intensity of $S_u(3,1)$ increases significantly while that of neighboring modes decreases due to the injection as shown in Fig. S10(a). This selective excitation of $S_u(3,1)$ is accompanied by a clear correlation peak, confirming the onset of synchronization. In contrast, for slightly detuned values such as $\Delta\nu = -1.1\,\text{GHz}$ or $\Delta\nu = 1.9\,\text{GHz}$, the spectral alignment is lost and the mode $S_u(3,1)$ is less intense, which goes along with a decrease in correlation.

A similar behavior is observed near $\Delta\nu \approx 80\,\text{GHz}$, where spectral alignment occurs between the slave mode $S_u(2,1)$ and the master mode $M_u(0,1)$. The intensity of the slave mode increases like in the first example, indicating its enhanced excitation due to spectrally aligned injection, and this results in a secondary synchronization peak. These observations demonstrate that the enhancement of a certain transverse mode of the slave laser, driven by spectral alignment with a strong mode of the master, plays a central role in enabling synchronization, but matching the spatial profiles of the involved master and slave modes is not required.

E. Correlation evolution with low- and high-pass filters

The preferential synchronization of low-frequency components appears to depend on the system and on the specific synchronization region. As shown in Fig. 2(d) of the main article, the correlation value increases as the high-frequency components are progressively removed, thereby revealing one of the characteristic timescales that favors higher correlation value.

Here, we present the evolution of the correlation values between the master and slave lasers for three correlation peaks, associated with the second injection experiment shown in Fig. 3. We determine how the correlation

evolves when applying a low- or high-pass filter, with the corresponding results displayed in Fig. S11.

These measurements of the evolution of the correlation value under low-pass and high-pass filtering show that, for the first two correlation peaks (a) and (b), the correlation increases as the high-frequency components are removed (Fig. S11 (a_2), (b_2)). The region corresponding to the third peak (c), at a detuning of 115 GHz, exhibits a lower synchronization level, associated with faster timescales. This ability to easily synchronize low-frequency components is consistent with the literature [S8-S11] and with the majority of our experiments.

REFERENCES

[S1] Bittner, S. & Sciamanna, M. Complex nonlinear dynamics of polarization and transverse modes in a broad-area vcsel. *APL Photonics* **7**, 126108 (2022).

[S2] Martin-Regalado, J. *et al.* Polarization properties of vertical-cavity surface-emitting lasers. *IEEE Journal of Quantum Electronics* **33**, 765–783 (1997).

[S3] Willemsen, M. B. *et al.* Polarization Switching of a Vertical-Cavity Semiconductor Laser as a Kramers Hopping Problem.*Physical Review Letters* **82**, 4815-4818 (1999).

[S4] Virte, M. *et al.* Deterministic polarization chaos from a laser diode. *Nature Photonics* **7**, 60–65 (2013).

[S5] Debernardi, P. *et al.* Influence of anisotropies on transverse modes in oxide-confined vcsels. *IEEE Journal of Quantum Electronics* **38**, 73–84 (2002).

[S6] Martìn-Regalado, J., Balle, S. & Miguel, M. S. Polarization and transverse-mode dynamics of gain-guided vertical-cavity surface-emitting lasers. *Optics Letters* **22**, 460–462 (1997).

[S7] Mercadier, J. *et al.* Chaos from a free-running broad-area VCSEL. *Optics Letters* **50**, 796–799 (2025).

[S8] Virte, M., Sciamanna, M. & Panajotov, K. Synchronization of polarization chaos from a free-running VCSEL. *Optics Letters* **41**, 4492-4495 (2016).

[S9] Boccaletti, S. *et al.* The synchronization of chaotic systems. *Physics Reports* **41**, 1-2 (2002).

[S10] Mercadier, J. *et al.* Optical chaos synchronization in a cascaded injection experiment. *Optics Letters* **49**, 2613-2616 (2024).

[S11] Hramov, A. E. and Koronovskii, A. A. Time scale synchronization of chaotic oscillators. *Physica D* **206**, 3-4 (2005).
